# Defining early surgery in traumatic spinal cord injury: admission-based versus injury-based timing

**DOI:** 10.1186/s42466-026-00470-y

**Published:** 2026-02-09

**Authors:** Audai H. Abudayeh, Iakiv Fishchenko

**Affiliations:** 1 Department of Traumatology and Orthopedics, Bogomolets National Medical University, Kyiv, Ukraine; 2 Department with Spinal (Neurosurgical) Center, SI Institute of Traumatology and Orthopedics of the National Academy of Medical Sciences of Ukraine, Kyiv, Ukraine

Dear Editor,

We read with great interest the study by Kamradt et al. [1] analyzing patterns and determinants of spine surgery timing in traumatic spinal cord injury (tSCI) using the TraumaRegister DGU^®^. The authors should be congratulated for assembling a large, multicenter cohort and for transparently discussing the inherent limitations of registry-based research. Their analysis provides valuable insight into real-world trauma system performance across German-speaking countries. We would like to comment on one methodological aspect that is central to the scientific interpretation of the findings, particularly regarding the concept of “early” surgery. In the present study, early surgery is defined pragmatically as intervention on the calendar day of hospital admission (day 0), with late surgery defined as day ≥ 1 [1]. The authors appropriately acknowledge that this classification does not correspond exactly to a 24-hour injury-based threshold and that a subset of surgeries categorized as “late” still occurred within 24 h. We fully recognize that the TraumaRegister DGU^®^ may not provide reliable injury-to-operation timestamps across the entire study period, and that registry constraints often necessitate such pragmatic definitions. However, this distinction has important scientific implications. The concept of early decompression in spinal cord injury—both biologically and within clinical guidelines—is defined relative to time from injury, not time from admission [2–4]. Injury-to-decompression time represents the hypothesized window of time-dependent neuroprotection, whereas calendar-day-of-admission surgery primarily reflects logistical and system-performance factors, including prehospital delay, interhospital transfer, and time-of-day effects. As a result, patients decompressed 2 h and 20 h after injury may both be classified as “day 0,” while patients decompressed at 22–23 h post-injury but after midnight may be classified as “late.” This form of exposure misclassification is not trivial and becomes particularly relevant when the manuscript interprets the high proportion of day-0 surgeries (approximately 69%) as reflecting adherence to recommendations for timely decompression and further infers a peak operative window likely within 0–12 h post-injury [1]. While such an inference is plausible in a high-functioning trauma system, it cannot be directly verified using an admission-day–based metric and should therefore be interpreted with caution. High-quality guideline syntheses support this cautious approach. The AOSpine clinical practice guideline on the timing of decompressive surgery in acute spinal cord injury explicitly rates the quality of evidence as low and the strength of recommendation as weak, despite generally favoring early intervention as an option [2]. This grading reflects heterogeneity across injury patterns and outcomes, and underscores the importance of precise exposure definitions when interpreting observational timing data. Likewise, landmark prospective cohorts and pooled individual-patient analyses that report associations between earlier surgery and improved neurological recovery define timing relative to injury and assess outcomes at longer-term follow-up, rather than relying on admission-based calendar thresholds [3–5].

Importantly, this methodological point does not diminish the value of the present study. On the contrary, the authors convincingly demonstrate that spine surgery is frequently performed rapidly after hospital admission and identify patient- and injury-related factors associated with delayed intervention—findings that are highly relevant for trauma system evaluation and quality improvement [1]. We suggest, however, that “day 0 surgery” be interpreted and described primarily as a system and workflow metric, rather than as a surrogate for evidence-based early decompression in the neuroprotective sense. Where injury-based timing is unavailable, we encourage careful terminology, avoidance of equivalence with ≤ 24-hour post-injury decompression, and—where feasible—sensitivity analyses using admission-to-surgery intervals in hours or restricted subsets with available time-of-day data. Such refinements would enhance comparability with the existing literature and strengthen the scientific precision of conclusions regarding surgical timing and neurological recovery.

## Data Availability

Not applicable.
